# Is there a response shift in generic health-related quality of life 6 months after glioma surgery?

**DOI:** 10.1007/s00701-016-3040-9

**Published:** 2016-12-07

**Authors:** Asgeir Store Jakola, Ole Solheim, Sasha Gulati, Lisa Millgård Sagberg

**Affiliations:** 1 Department of Neurosurgery, Sahlgrenska University Hospital, Blå Stråket 5, vån 3, 413 45 Göteborg, Sweden; 2 Institute of Neuroscience and Physiology, Sahlgrenska Academy, University of Gothenburg, Gothenburg, Sweden; 3 Department of Neurosurgery, St. Olavs Hospital, Trondheim, Norway; 4 Norwegian Advisory Unit for Ultrasound and Image-Guided Therapy, St. Olavs University Hospital, Trondheim, Norway; 5 Department of Neuroscience, Norwegian University of Science and Technology, Trondheim, Norway; 6 Norwegian Centre of Competence in Deep Brain Stimulation for Movement Disorders, St. Olavs University Hospital, Trondheim, Norway

**Keywords:** Glioma, Neurosurgery, Quality of life, Brain neoplasm, Surgical management

## Abstract

**Background:**

Patients may recalibrate internal standards when faced with a serious diagnosis or neurological deficits. This so-called response shift is important to understand in longitudinal health-related quality of life (HRQoL) data, but this is not quantitatively assessed in glioma patients.

**Methods:**

Patients with gliomas were eligible for this HRQoL study. We used EuroQol-5D 3 L to assess generic HRQoL with assessment preoperatively and at 6 months postoperatively. At time of follow-up, patients scored how they considered their baseline HRQoL in retrospect using the same questionnaire (“then-test”).

**Results:**

Seventy-three patients were enrolled between January 2013 and September 2015. With the then-test approach, the mean EQ-5D 3 L index was similar compared to baseline (0.77, mean difference 0.01, 95% CI −0.57 to 0.07, *p* = 0.82). Also, then-test and baseline VAS score were similar (mean difference 0, 95% CI −7 to 7, *p* = 0.97). However, a 0.10–0.13 difference from baseline was observed in patients that improved or deteriorated in HRQoL at follow-up according to the then-test EQ-5D 3 L index value. The direction of change as observed from the then-test was similar to the direction of clinical change, reducing the impact of any HRQoL change from baseline to follow-up.

**Conclusions:**

On average, we observed no response shift using EQ-5D 3 L in the selection of glioma patients able to participate at 6 months after surgery. However, following change in HRQoL at follow-up, response shift seems to reduce the effects of HRQoL changes by lowering of internal standards in patients that deteriorate and raising the standards in patients that improve.

## Introduction

Research on health-related quality of life (HRQoL) in glioma patients is gaining interest [14, 18, 22]. However, so-called response shift is frequently considered to interfere with interpretation of longitudinal HRQoL data [11, 14, 18]. Response shift refers to the phenomenon where patients score better because over time they adapt to a new situation; for instance, a handicap or an illness [17, 18]. It is considered to involve elements of recalibration of internal standards, change in priorities and/or a different view upon the concept of HRQoL [3, 8, 27]. In patients with gliomas all these elements may change when faced with neurological deficits or being diagnosed with a life-threatening illness [18].

Thus, such recalibration may therefore affect longitudinal HRQoL data and interpretation of results, but unless assessed directly it is impossible to detect if a recalibration has occurred [3]. The most common approach to study response shift is the so-called “then-test approach” where respondents retrospectively score how they at time of follow-up consider their earlier or baseline HRQoL in the light of their new situation [17, 25, 27].

In a meta-analysis from 2006, the effect sizes of response shift were found to be small, with the largest effect sizes detected for fatigue and global HRQoL [25]. Even though response shift is known to occur in cancer patients [8, 9, 17, 18], we have not found response-shift studies in glioma patients. Searching for clinically relevant subgroups that could exhibit response shift is also indicated.

In this project we aimed to prospectively study the direction and magnitude of response shift in glioma patients using a global, generic HRQoL measure. Further, since response shift is most pronounced in the presence of a trigger [2] (i.e. less likely to occur in patients with stable condition), we explore response shift in patients with a significant HRQoL change at follow-up.

## Materials and methods

### Study population

**Fig. 1:**
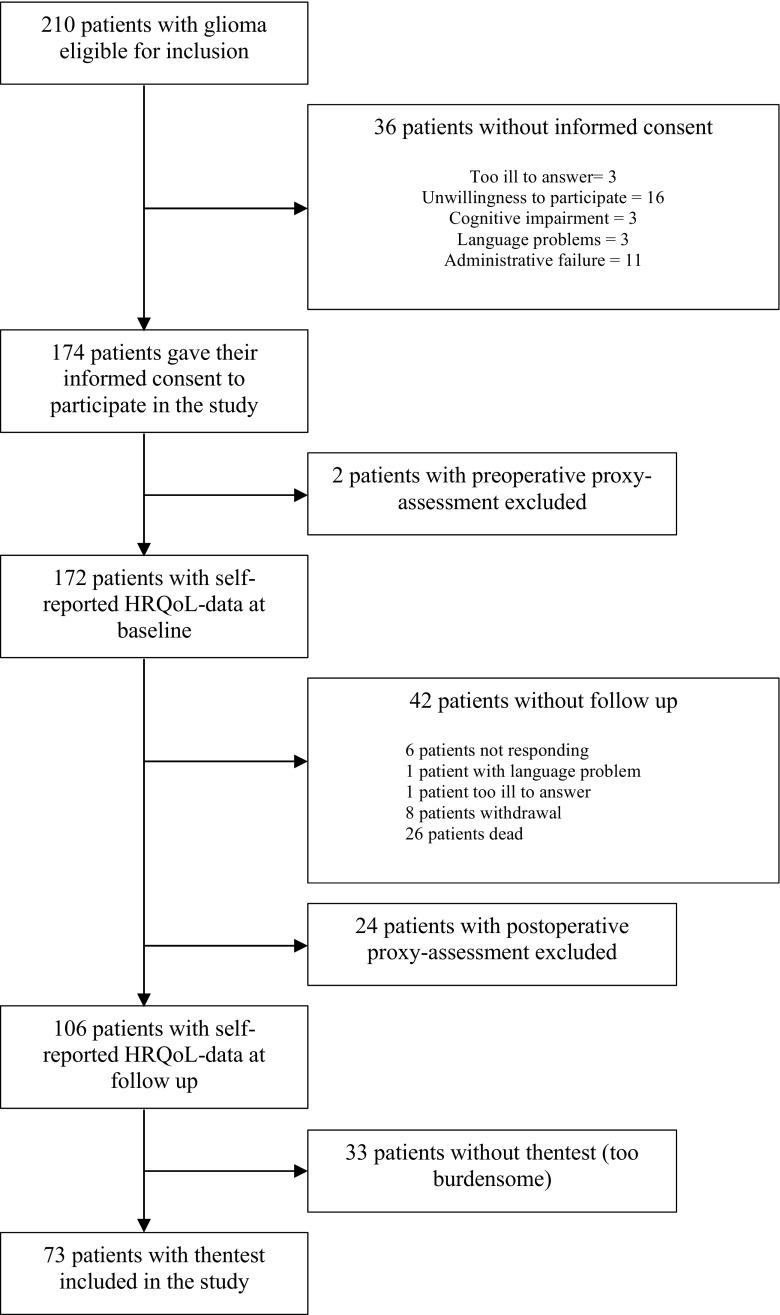
Flow chart

All adult patients (≥18 years old) that underwent surgery for glioma at St. Olavs University Hospital, Trondheim, Norway, in the period from January 2013 through September 2015 were eligible for inclusion in this study. In this period 210 patients with glioma underwent a neurosurgical procedure of biopsy or resection. As shown in the flow-chart (Fig. 1), we included in total 73 patients that had both a self-reported baseline assessment and a self-reported postoperative assessment at 6 months with the renewed retrospective scoring of their baseline HRQoL.

All included patients had histopathological diagnosis of WHO grade II-IV glioma confirmed by a neuropathologist according to the 2007 WHO classification [19].

### Euroqol 5D 3L

The EuroQol 5D 3L (EQ-5D 3L) is a generic measure of health-related QoL developed by the EuroQol Group [28]. The EQ-5D 3L has been validated in a Norwegian normal population [21]. The questionnaire has been applied to a wide range of health conditions and treatments.

In EQ-5D 3L, five dimensions of HRQoL are scored: mobility, self-care, usual activities, pain/discomfort and anxiety/depression, with three possible answers to each dimension, i.e. ‘no problem’, slight problem’ or ‘major problem’. This results in the 243 different possible health states, which are transformed into an index value based on a large survey in the UK population [6]. EQ-5D 3L index value is from –0.594 to 1, where 1 corresponds to perfect health, and 0 to death. Negative values are considered to be worse than death. The questionnaire also contains a visual analogue scale (VAS), reflecting the global health state from 0 (worst possible score) to 100 (best possible score).

We chose to use EQ-5D 3L due to the simplicity of the instrument, to enhance patient perception and perhaps also compliance. We have previously demonstrated that EQ-5D 3L index value shows good correlation to Karnofsky Performance Scale (KPS) in patients with gliomas and is responsive to new neurological deficits which is highly relevant in this patient group [15]. Further, we have found the minimal clinically important change (MIC) to be approximately 0.13–0.15 [22]. In this study we decided a priori that 0.15 was the minimum magnitude of change necessary to be a clinical important change.

### Data collection

Our routine has been that patients provided written informed consent and filled out the EQ-5D 3L questionnaire 1–3 days before surgery. The operating surgeon scored preoperative KPS prospectively on admission. Patient follow-up by a study nurse was scheduled at 6 months postoperatively to allow recovery from transient surgically induced deficits. At 6 months, patients undergoing radiotherapy had also had time for recovery; however, tumour progression/recurrence may, on the other hand, occur.

Data were collected from the hospital’s electronic patient charts. Patient characteristics and preoperative status including Charlson comorbidity index [5], KPS [16] and eloquence in tumour location [24] were registered. Complications were scored according to the classification system proposed by Landriel Ibañez and co-workers [10]. Tumour volumes and resection grades were determined from preoperative and early postoperative magnetic resonance imaging (MRI) volumes using an ellipsoid model (4л × r^3^/3) where gross total resection (GTR) was defined as <0.175 cm^3^ residual tumour tissue on the early (<72 h) postoperative 1.5-T or 3.0-T MRI scans; both techniques previously described by others [26]. If the tumour had a smaller contrast-enhanced region being surrounded by a larger region with T2/FLAIR abnormalities believed to be glioma and not oedema the entire tumour was measured, and not only the contrast-enhancing part. For non-contrast-enhancing gliomas, the T2/FLAIR images were used for volume assessment.

### Response shift

To assess response shift, we used a pre-test/post-test design with a then-test [29]. The patients were asked to score their baseline ratings of the five EQ-5D 3L domains and the VAS score immediately after the post-test assessment at 6 months follow-up. The research nurse emphasised that the intention of the retrospective test was not to remember and copy their answers at baseline but to provide a renewed baseline HRQoL as they now would consider it in retrospect (then-test). According to this method, patients use their new internal standards in the then-test. The mean difference between the then-test and prospective baseline test was then calculated to provide the recalibration response shift effect, while the mean difference between the then-test and the post-test was calculated to provide a better estimate of the adjusted time effect (i.e. the true change).

### Statistical analysis

To analyse data and to create graphs we used the software package SPSS (version 21.0; SPSS, Chicago, IL, USA). Q-Q plots were used to test if data were normally distributed. When analysing changes in EQ-5D 3L (e.g. before and after surgery) a paired sample *t*-test was used. Comparisons of continuous data were done with independent samples *t*-test. Categorical data were analysed with Pearson’s chi-squared test. A *p* value ≤0.05 was considered statistically significant.

## Results

Baseline and surgical characteristics are summarised in Table 1. In 43 patients (59%) the surgery was a primary operation. The histopathology revealed a diffuse low-grade glioma in 26 patients (36%) and a high-grade glioma in 47 patients (64%).

**Table 1:** Baseline characteristics

	*n* = 73
Age in years, mean (SD)	49 (15)
Female, *n* (%)	26 (36)
Preoperative KPS ≥70, *n* (%)	70 (96)
Preoperative CCI >1, *n* (%)	3 (4)
Symptoms, *n* (%)	
Headache	24 (33)
Seizures	27 (37)
Cognitive	17 (26)
Dysphasia	6 (8)
Motor	3 (4)
Primary surgery, *n* (%)	43 (59)
Preoperative use of corticosteroids, *n* (%)	28 (38)
Preoperative tumour volume, median (IQR)	24.2 (5.7–43.9)
Eloquent, *n* (%)	21 (29)
Resection, *n* (%)	70 (96)
Histopathology, *n* (%)	
WHO grade II	26 (36)
WHO grade III	19 (26)
WHO grade IV	28 (38)
Extent of resection, median (IQR)	93.2 (81.7–100)
Baseline EQ-5D 3 L index value, mean (SD)	0.78 (0.24)
Baseline EQ-VAS, mean (SD), *n* = 67	73 (21)

*KPS* Karnofsky Performance Scale score, *CCI* Charlson comorbidity index, *Eloquent* refers to Sawaya grade 3

Follow-up data at 6 months from baseline are presented in Table 2. Mean EQ-5D 3 L index value and VAS score at follow-up did not differ from the baseline scores (*p* = 0.52 and *p* = 0.94, respectively). According to the suggested glioma MIC value for the EQ-5D 3L index [22], we observed that 14 (19%) improved, 43 (59%) remained unchanged and 16 (22%) deteriorated from the true baseline test.

**Table 2:** Important variables 6 months postoperatively

	*n* = 73	Significant change from baseline
6 month KPS ≥70, *n* (%)	65 (89)	*p* = 0.21
EQ-5D 3 L index value at 6 months, mean (SD)	0.79 (0.21)	*p* = 0.52
MIC group, *n* (%)		NA
Better	14 (19)	
Similar	43 (59)	
Worse	16 (22)	
VAS score at 6 months, mean (SD), *n* = 68	72 (17)	*p* = 0.94

*MIC* minimal clinically important change; the MIC is set to 0.15, as reported by Sagberg and co-workers

Using the then-test approach on the entire sample, we found that the mean EQ-5D 3L index was similar at then-test compared to baseline (0.77, mean difference 0.01, 95% CI −0.57 to 0.07, *p* = 0.82, Fig. 1). Similarly, then-test and baseline VAS score was compared demonstrating a mean difference of 0 (95% CI −7 to 7, *p* = 0.97). Since 30 patients had been operated on previously, we assessed if there was any difference in response shift using EQ-5D 3L index between groups, but there was none (mean difference 0.06, 95% CI −0.08 to 0.21, *p* = 0.40). Using the then-test to evaluate change in relation to MIC, we observed that 21% improved, 54% remained unchanged and 26% deteriorated. Thus, in this sample of glioma patients, we observed *on average* no indication of any statistically significant or clinically relevant response shift.

### Patients with minimal clinical important change

We found that the 16 patients who reported clinically significant deterioration in EQ-5D 3L index at 6 months compared with true baseline values reported a difference of 0.13 in the then-test versus true baseline, a result that indicate that their baseline HRQoL was considered to be worse when scored in retrospect. However, in these patients the difference between the true VAS at baseline and the then-test VAS was 0.

When analysing those who improved (*n* = 14) at 6 months compared to true baseline, they had a 0.10 difference in then-test compared to true baseline, with the direction that indicated that they considered their baseline HRQoL to be better in retrospect.

**Fig. 2:**
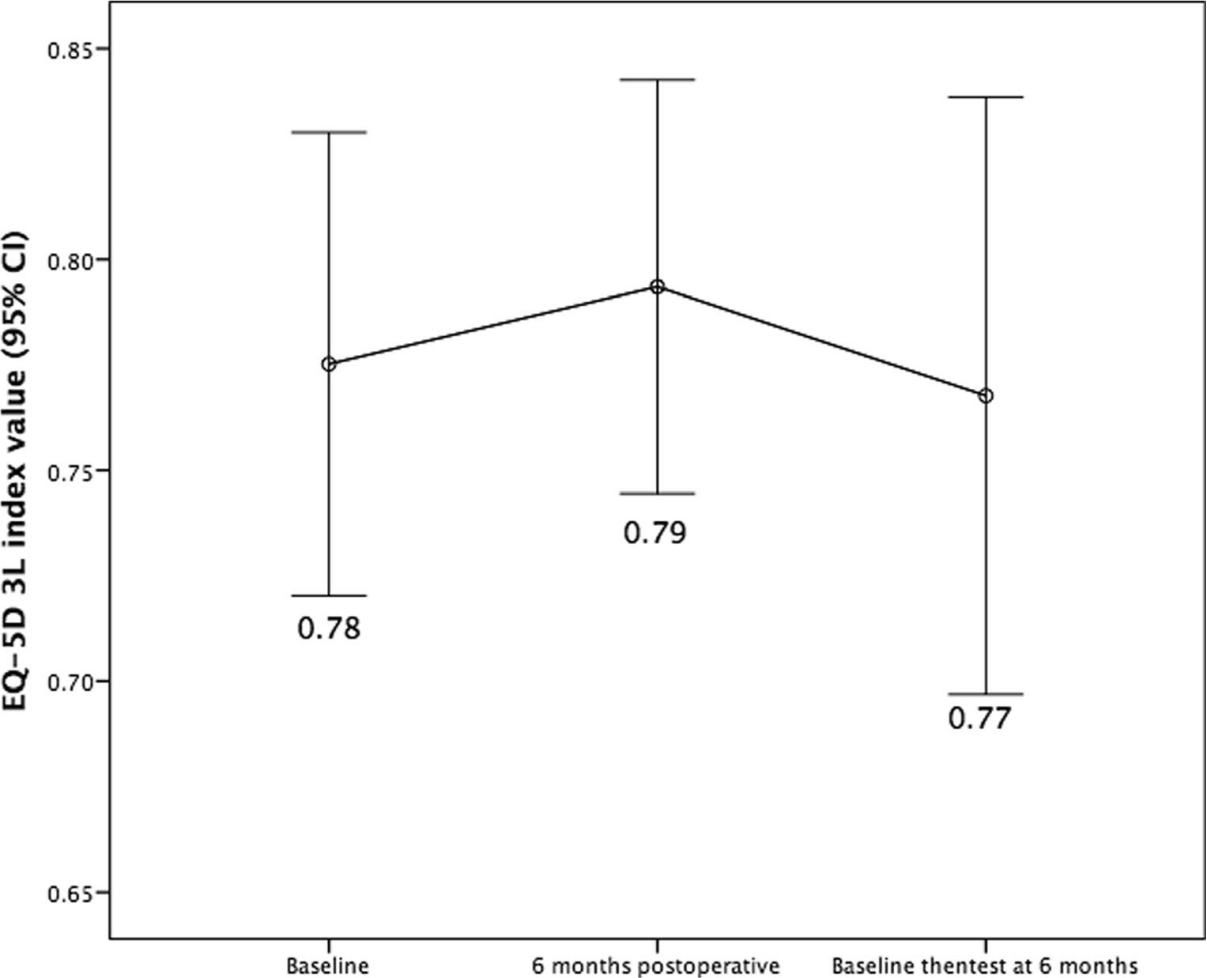
Response shift assessed in the entire sample (*n* = 73)

**Fig. 3:**
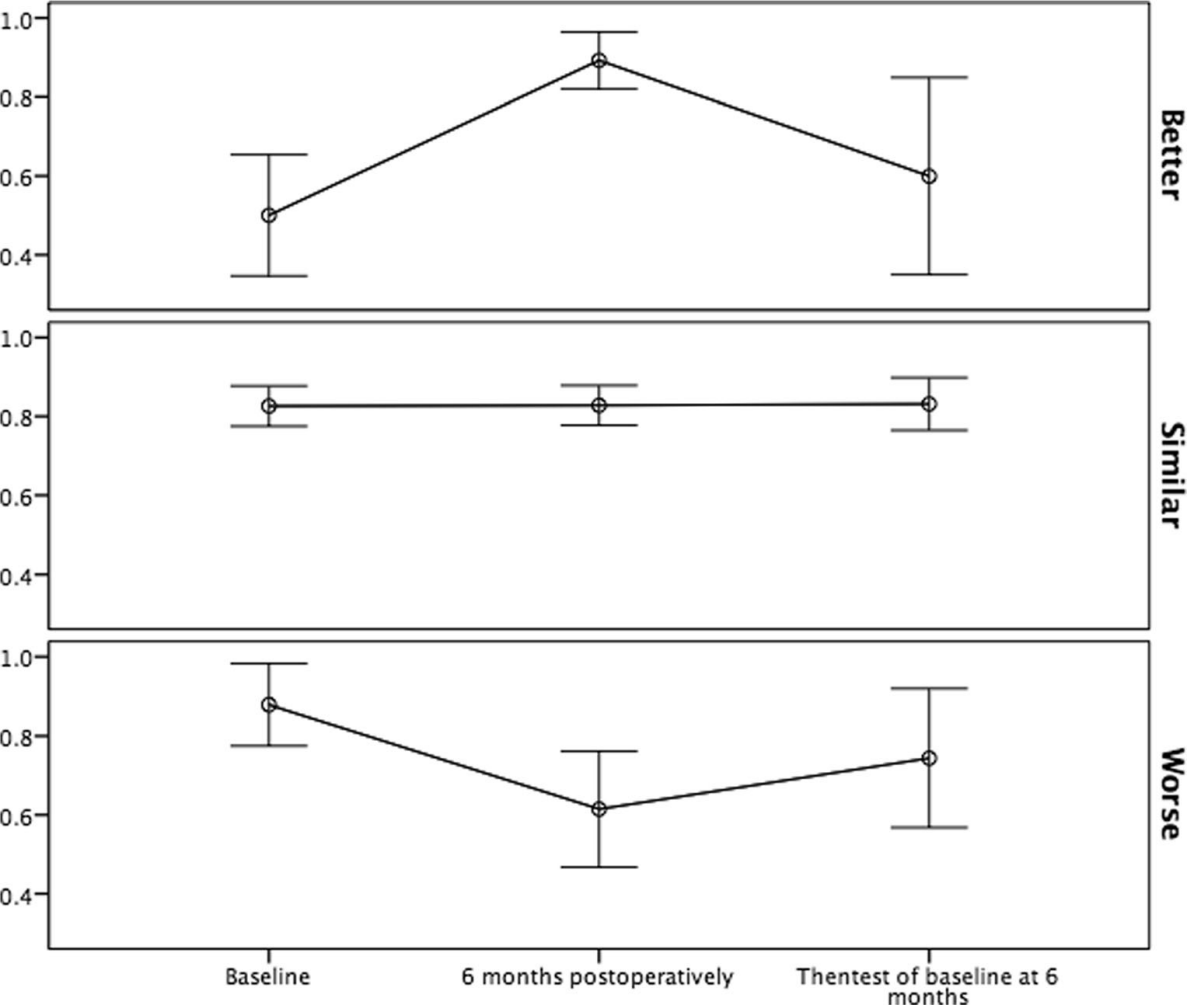
Response shift seen in EQ-5D 3L index value in patients according to groups based on minimal clinically important change

Similarly, the VAS score was considered 6 points better in then-test than at baseline. In Table 3 we also explored the different EQ-5D 3L domains at then-test compared with baseline assessment. The findings of EQ-5D 3L index value in the different MIC groups are visualised in Figs. 2 and 3 to enhance interpretation.

**Table 3:** How the then-test of EQ-5D domains compare with baseline assessment

	Better	Similar	Worse
ENTIRE SAMPLE (*n* = 73)			
Then-test mobility (*n* = 72)	3	67	2
Then-test self-care (*n* = 72)	2	69	1
Then-test activity (*n* = 73)	12	50	11
Then-test pain (*n* = 70)	14	46	10
Then-test anxiety (*n* = 72)	12	49	11
MIC IMPROVED (*n* = 14)			
Then-test mobility (*n* = 14)	1	12	1
Then-test self-care (*n* = 14)	2	11	1
Then-test activity (*n* = 14)	2	10	2
Then-test pain (*n* = 14)	2	10	2
Then-test anxiety (*n* = 14)	7	5	2
UNCHANGED (*n* = 43)			
Then-test mobility (*n* = 43)	1	42	0
Then-test self-care (*n* = 43)	0	43	0
Then-test activity (*n* = 43)	10	26	7
Then-test pain (*n* = 41)	11	27	3
Then-test anxiety (*n* = 42)	3	33	6
MIC DETERIORATED (*n* = 16)			
Then-test mobility (*n* = 15)	1	13	1
Then-test self-care (*n* = 15)	0	15	0
Then-test activity (*n* = 16)	0	14	2
Then-test pain (*n* = 15)	1	9	5
Then-test anxiety (*n* = 16)	2	11	3

Better in this context refer to that the then-test indicate fewer symptoms/problems than the baseline assessment

### Exploratory subgroup analyses of patients with clinically significant response shift

We used the suggested MIC as guidance in defining patients who significantly considered their baseline to be better in retrospect (*n* = 18) and those who significantly considered their baseline to be worse in retrospect (*n* = 15).

We searched predictors among the variables in Table 1 in addition to significant surgical complications (Landriel Ibañez grade 2 or more) or surgically induced neurological deficits.

Of patients who scored their baseline better in retrospect compared to the rest of the cohort, only seizures at baseline (61% versus 29%, *p* = 0.015) and baseline EQ-5D 3L index value (0.63 versus 0.82, *p* = 0.003) were factors associated with significant response shifts.

Of patients who scored their baseline worse in retrospect compared to the rest of the cohort, only significant surgical complications was an associated factor (27 versus 3%, *p* = 0.004).

## Discussion

Response-shift assessment of baseline EQ-5D 3L index value and VAS score at 6 months following surgery for glioma patients using the then-test approach revealed that in our selection of patients there was on average no response shift. This may be due to the fact that the vast majority of our respondents were clinically stable at follow-up as assessed with EQ-5D 3L. Another factor contributing to averaging of results was that we had almost similar proportion of patients improving (19%) and deteriorating (22%) in HRQoL at 6 months from baseline. Response shifts were more frequently observed in both patients that improved and patients that deteriorated according to the MIC, but the direction was opposite. Patients that improved had raised their internal standards, while patients that deteriorated had lowered their internal standards.

Further, we explored possible factors associated with significant response shift. A low baseline HRQoL is likely to be considered better in retrospect according to these exploratory analyses. This may possibly relate to ceiling effects of generic HRQoL measures at baseline, where improvement of maximum score is impossible, or simply due to regression of the mean [22]. Also, patients with seizures at baseline were over-represented in the group that reported better baseline HRQoL in retrospect. These patients are likely to have improved HRQoL at follow-up due to chance of seizure freedom with extensive surgery, and therefore now perhaps forgot or repressed how it was living with seizures [4].

As observed by others studying other conditions, response shift is only present in case of a catalyst [2], and in this regard it is apparently not enough to be diagnosed with a glioma or facing repeated surgery due to recurrence, as we only observed response shift in relation to significant changes in HRQoL after surgery. However, we observe that significant complications triggered a response shift in terms of considering the baseline HRQoL worse in retrospect, reducing the negative impact on HRQoL from the surgical complications 6 months after surgery.

As seen above, the direction of response shift in our study suggests that response shift may reduce the actual changes seen in longitudinal HRQoL studies, with respect to both deterioration and improvement, when using EQ-5D 3L index value according to the then-test model [25]. One practical implication this has for interpretation is that glioma patients who remain stable after surgery according to MIC groups are not stable simply due to response shift since we did not observe any response shift in this stable group. Further, for glioma patients with significant changes in HRQoL after surgery, they seemingly more frequently recalibrate their internal standards, reducing the actual effect size of the change as observed in longitudinal studies.

Our study was not designed to evaluate the relative importance of the different elements of response shift (i.e. recalibration, reprioritisation and reconceptualisation). In a recent study of patients with prostate cancer, reconceptualisation was not an important factor, perhaps indicating that the other two are more important elements in response shift among cancer patients [8]. Others consider the elements of reprioritisation and reconceptualisation not to be a true response shift, but rather coping strategies affecting the true value [3]. In this view, recalibration is the only true response shift.

The underlying assumptions of the then-test used in this study have recently been criticised [27]. Most importantly, the assumption of cognitive consistency of respondents at the different time points may not hold true [27]. Also, recall bias of previous health condition is a concern [25, 27], especially since this may be more pronounced in patients with cognitive deficits [1]. In our study, all patients were able to complete the then-test, but many were naturally cognitively impaired. This may contribute to the rather wide confidence intervals observed in this study [3]. Even though global HRQoL has been found to have among the larger effect sizes, generic HRQoL measures are in general less sensitive and this may have contributed to the group level results in our study [25]. Also, since response shift assessments require patients that are able to report their own health state at both baseline and at follow-up, thus terminally ill patients, patients with severe cognitive deficits or patients with severe language problems were not included in this study. Consequently, the interpretation of our results must be understood in the light of the selection reported in Fig. 1, where an unavoidable selection bias seems to be present which may influence our results. Finally, in the light of the above-mentioned limitations and since this is the first paper to assess response shift after glioma surgery caution is needed when interpreting our results, and especially the more exploratory findings should be considered hypothesis-generating.

This study is important for the interpretation of our earlier studies using EQ-5D 3L in gliomas [7, 12–15, 22, 23]. Most importantly, we are now more confident that it is not a response-shift artefact when patients reported stable HRQoL. It is perhaps comforting that the response shift may reduce the negative effect of glioma surgery or disease progression, but we should not accept that patients reduce their standards if this can be avoided with either safer surgery or more effective treatment delaying time to progression. Thus, we should continue to evaluate our results and readjusting our practice if this improves the onco-functional balance [20].

## Conclusions

Our results indicate that response shift in glioma patients undergoing surgery is dependent on changes in HRQoL at time of assessment. Importantly, patients reporting stable HRQoL at follow-up demonstrated no response shift in our study. Further, response shift seems to reduce the effects of HRQoL changes by lowering of internal standards in patients that deteriorate and raising the standards in patients that improve.
